# Beyond stress response: OST1 opening doors for plants to grow

**DOI:** 10.1007/s44154-022-00069-8

**Published:** 2022-10-25

**Authors:** Leelyn Chong, Rui Xu, Lixia Ku, Yingfang Zhu

**Affiliations:** 1 State Key Laboratory of Crop Stress Adaptation and Improvement, School of Life Sciences, Henan University, Kaifeng, 475001 China; 2 College of Agronomy, Synergetic Innovation Center of Henan Grain Crops and National Key Laboratory of Wheat and Maize Crop Science, Henan Agricultural University, Zhengzhou, 450046 China

**Keywords:** Abiotic stress, OST1/SnRK2.6, Abscisic acid (ABA), Flowering, Growth, Development

## Abstract

The sucrose non-fermenting 1 (SNF1)-related protein kinase 2 (SnRK2) family members have been discovered to regulate abiotic stress response via the abscisic acid (ABA)-independent and dependent signaling pathways. SnRK2.6, also known as Open Stomata 1 (OST1), is a serine/threonine protein kinase that plays critical roles in linking ABA receptor complexes and downstream components such as transcription factors and anion channels to regulate stress response. Asides from its well-known regulatory roles in stomatal movement and cold stress response, OST1 has also been demonstrated recently to modulate major developmental roles of flowering and growth in plants. In this review, we will discuss about the various roles of OST1 as well as the ‘doors’ that OST1 can ‘open’ to help plants perform stress adaptation. Therefore, we will address how OST1 can regulate stomata apertures, cold stress tolerance as well as other aspects of its emerging roles such as balancing flowering and root growth in response to drought.

## Introduction

Environmental stresses simultaneously affect all parts of plant cells. In order to survive and reproduce, plants must perceive these stress signals and pass them within cells as well as between cells and tissues (Zhang; et al., 2022). Depending on the signal, plants may perform growth and development adjustment. It has been known that plants possess various mechanisms to regulate stress response; and the abscisic acid (ABA) signaling pathway is one of the key strategies that plants utilize to manage stress response (Gong; et al., 2020a; Zhu, 2016). Environmental stress such as drought severely impedes plant growth and development since drought causes osmotic stress to plants which further places plant cells in dehydration mode. The ABA signaling plays a significant role for plants to induce drought stress response and tolerance because ABA is produced under osmotic stress conditions (Chen; et al., 2020). To date, ABA signaling is known to be mediated by a perception-transduction-response pathway which consists of pyrabactin resistant/pyrabactin resistant-like/regulatory component of ABA responses (PYR/PYL/RCAR) receptors, clade A type 2C protein phosphatases (PP2Cs), and SNF1-related protein kinase 2 (SnRK2) (Chong; Guo; Zhu, 2020; Hou; et al., 2016; Umezawa; et al., 2010).

Since the last decade, SnRK2s have been viewed as one of the main regulators of ABA signaling. There are three subclasses (I, II, III) of SnRK2s mediated by ABA receptors and protein phosphatases. They are categorized based on their amino acid sequence similarity and their ability to trigger ABA response. SnRK2 protein kinases can mediate stress responses via either or both the ABA-dependent or ABA-independent pathway (Cutler; et al., 2010; Fujii; Zhu, 2012). Thus far, it is known that osmotic stress can activate all SnRK2 family members except Arabidopsis SRK2J/SnRK2.9 (Subclass I). Subclass I SnRK2s are thereby categorized as ABA non-responsive whereas subclasses II and III are characterized as ABA-responsive since subclasses II and III SnRK2s have been demonstrated to be activated by ABA. Upon stimulation of ABA, drought, or osmotic stress, SnRK2.2/2.3/2.6 s are activated in plants. SnRK2s have been shown to phosphorylate a component of the Target of Rapamycin (TOR) complex called Raptor (Wang; et al., 2018). TOR is a Ser/Thr kinase found in eukaryotic cells that plays a crucial role in cell metabolism and growth (Zhu; et al., 2022). This growth-promoting kinase TOR can inhibit ABA and osmotic stress responses under stress-free condition (Belda-Palazon; et al., 2020). This discovery is important because it reveals a reciprocal regulation between stress and plant growth programs in plants; and this presents one line of evidence that indicates SnRK2s can involve in plant stress response and growth.

In the situation without ABA, TOR kinase can phosphorylate PYR1/PYLs/RCARs and affect the receptors’ association with PP2Cs; which leads to SnRK2s inhibition. In the presence of ABA, PYR/PYL/RCARs are induced to interact with PP2Cs and SnRK2s are further activated in the ABA signaling pathway to transduce a stress response. Drought puts plants under osmotic stress and this stress signal can be transduced through either or both the ABA-independent and ABA-dependent pathway. Several research groups in 2020 had independently reported the kinases that directly phosphorylate and activate SnRK2s in response to osmotic stress in Arabidopsis (Katsuta; et al., 2020; Lin; et al., 2020; Lozano-Juste; Alrefaei; Rodriguez, 2020; Soma; et al., 2020). These kinases are called Raf (rapidly accelerated fibrosarcoma)-like kinases (RAFs). RAFs encode mitogen-activated protein kinase kinase kinases (MAPKKK/MAP3K) and they are characterized into different subfamilies. However, there is no direct evidence that shows RAFs can phosphorylate and activate MAPKK as MAPKKK yet. B2 and B3-RAF subfamily members activate subclass III SnRK2s favorably in the ABA-dependent pathway. Moreover, they stimulate the phosphorylation of downstream substrates to promote osmotic stress and ABA responses in plants. B4 RAF subfamily members also participate in osmotic stress but they mainly activate subclass I SnRK2s in the ABA-independent pathway. B4-RAFs phosphorylate subclass I SnRK2-VARICOSE (VCS) signaling pathway to regulate mRNA degradation for a stress response (Soma; et al., 2020). All of these findings have indicated that the SnRK2s family members play a significant role in regulating stress response. Additionally, RAF-activated SnRK2s can phosphorylate additional downstream targets, including other SnRK2s, to amplify ABA signals (Chong; Hsu; Zhu, 2022; Lin; et al., 2021).

SnRK2.6, also known as SRK2E and Open Stomata 1 (OST1), is a subclass III SnRK2. This protein kinase has consistently been reported to regulate ABA signaling and abiotic stress responses in plants (Nakashima; et al., 2009; Nakashima; Yamaguchi-Shinozaki, 2013; Yoshida; Mogami; Yamaguchi-Shinozaki, 2014). As of now, there is still not much known about how the phosphorylation cycle between OST1 and phosphatases is regulated in response to ABA. Apparently, a number of phosphorylation sites are present in OST1 and these sites can either be auto-phosphorylated or trans-phosphorylated by OST1-interacting proteins (Shang; et al., 2021). MAPKKK has been uncovered recently as an upstream component that can trans-phosphorylate and regulate OST1 activity in the presence of ABA (Lozano-Juste; Alrefaei; Rodriguez, 2020). Furthermore, it has diverse functions ranging from regulating stress tolerance, guard cell aperture, as well as reactive oxidative species (ROS) production. In 2022, OST1 was revealed that it could contribute to plant stress response through modulating flowering and root growth (Chen; et al., 2022; Chong; et al., 2022). In this review, we will provide a synopsis of OST1’s multifunctional roles in plants as well as its potential for ‘opening doors’ to perform stress response and help plants grow under stress.

## OST1 regulates stomatal movement in response to environmental stress

Stomatal pores in the plant epidermis regulate CO_2_ uptake for photosynthesis and transpiration (Engineer; et al., 2016; Pillitteri; Torii, 2012). The closing and opening of the pores are controlled by the two surrounding guard cells that can osmotically shrink and swell. In addition, these guard cells can integrate a number of environmental and endogenous signals to regulate stomatal aperture (Qi; et al., 2018). It is recognized that once the ABA receptors form a complex by binding to PP2Cs, SnRK2s as well as the membrane receptor-like protein kinase GUARD CELL HYDROGEN PEROXIDE-RESITANT 1 (GHR1) will get activated. The downstream targets of SnRK2s include SLOW ANION CHANNEL-ASSOCIATED 1 (SLAC1) and the R-/QUAC-type anion channel QUAC1. Meanwhile, the K^+^ inward rectifying channel (KAT1) is inactivated to trigger stomatal closure and decrease transpiration and photosynthesis (Bharath; Gahir; Raghavendra, 2021).

**Fig. 1:**
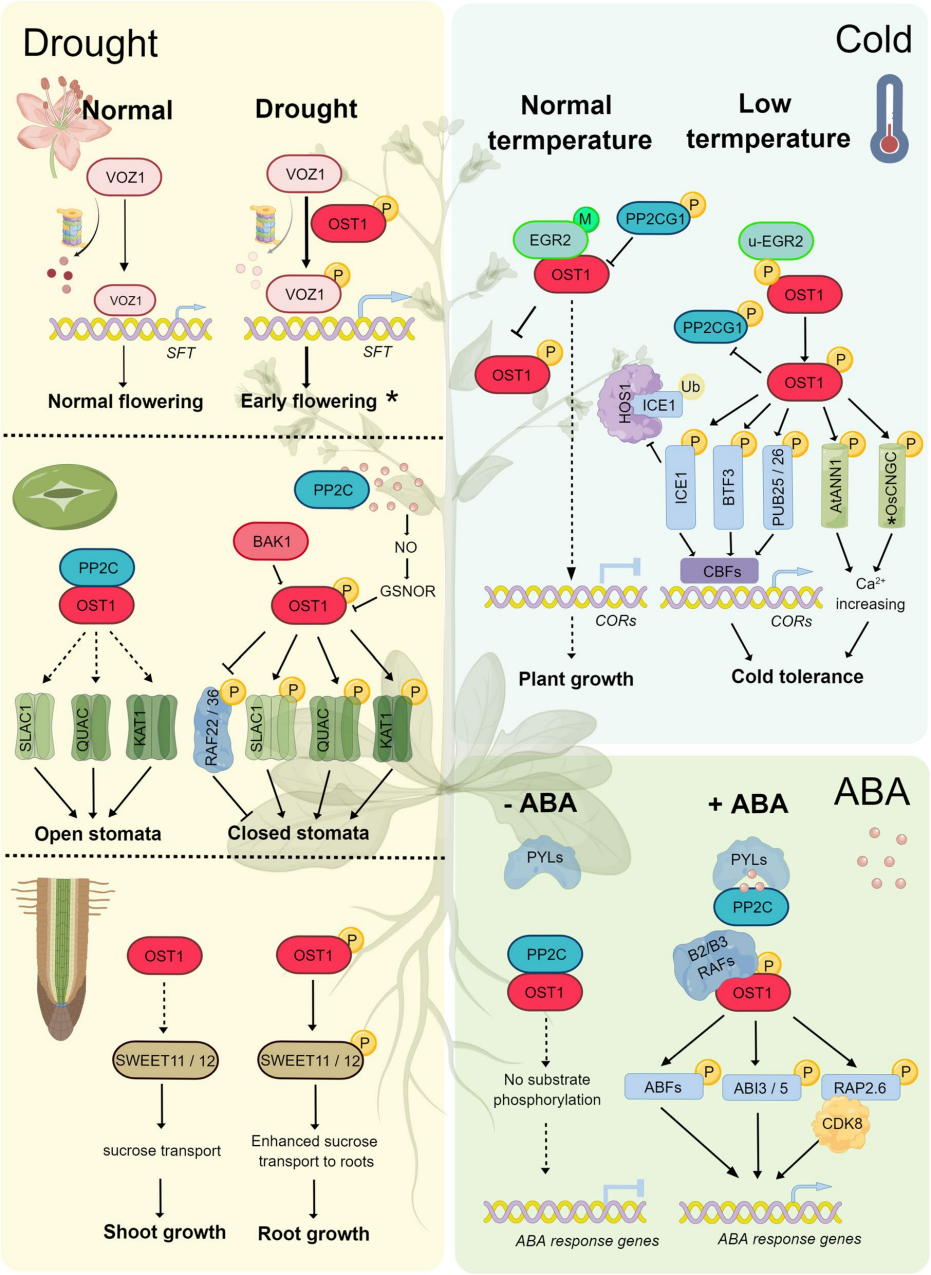
OST1’s multiple functional roles in plant stress response, growth, and development. OST1 is a critical member of the SnRK2 family. It performs various important roles in plants such as stomatal closure and cold stress response regulation. Furthermore, it can modulate plant growth and development such as balancing flowering and root growth in response to drought. Through phosphorylation/dephosphorylation, OST1 interacts and cooperates with other core members in the ABA signaling pathway to regulate TFs or bind with other proteins to form a module or a dynamic network that regulates downstream expression in the nucleus. In the absence of ABA, PP2Cs interact with OST1 and this inhibits OST1 from phosphorylating TFs that regulate stomatal closure. When ABA is present, ABA receptors and ABA-bound PYLs can recruit PP2C from OST1. B2/B3 RAFs can phosphorylate and activate OST1, which would further phosphorylate various TFs such as ABFs, ABI3/5 and RAP2.6. The mediator subunit CDK8 can facilitate the recruitment of RNA polymerase II to the promoter of TF target genes to regulate their transcription. Under normal temperature, EGR2 and PP2CG1 inhibit OST1 from activation through dephosphorylation. Upon cold stress, the binding of EGR2 and PP2CG1 to OST1 is impaired. Cold-activated OST1, however, interacts with and phosphorylates ICE1 and PP2CG1. It can also stabilize the protein stability of ICE1 by disrupting its association with the E3 ligase HOS1. Moreover, OST1 can phosphorylate U-box E3 ligases PUB25/PUB26 and BTF3s to promote the stability of CBF proteins under cold stress. Besides, OST1 can also phosphorylate the transporters ANNEXIN1 and CNGCs (in rice) to trigger the Ca^2+^ signaling in response to cold. Under the influence of ABA or drought, OST1 is activated from the inhibition by PP2C. OST1 then phosphorylate ion channels SLAC1, QUAC and KAT1 to control the ABA and stress induced stomata closure. BAK1 can also form a complex with OST1 to positively regulate the ABA-mediated stomata closure. Moreover, ABA can induce NO production, which in turn negatively regulates OST1 activity through GSNOR-mediated S-nitrosylation. Under normal condition, SlVOZ1 protein is not stable in tomato. Upon drought stress, SlOST1 is activated and interacts with and phosphorylates SlVOZ1, which leads to increased protein stability and nuclear accumulation of SlVOZ1. SlVOZ1 can directly bind to the *SFT* promoter to stimulate tomato flowering in response to drought stress. Under drought condition, OST1 and SnRK2.2/2.3 are activated and can phosphorylate SWEET11 and 12, resulting in sucrose transport from shoot to root, thus promoting root growth under drought stress. Figure 1 was created by Figdraw (https://www.figdraw.com/static/index.html#/)

The encoding gene of OST1 is expressed in guard cells and vascular tissues. OST1 can act upstream of ROS production as well as regulating stomatal closure triggered by ABA (Mustilli; et al., 2002). In fact, OST1 serves as the ABA receptor-coupled core signaling component in guard cells because it can perform phosphorylation on the downstream components of the ABA signaling pathway (Acharya; et al., 2013). Apparently, OST1 can act in the interval between ABA perception and ROS production. Furthermore, post-translational modifications (PTMs) have also been reported to control the function and activity of OST1. Persulfidation, S-nitrosylation and phosphorylation can interact to fine tune the activity of OST1 (Chen; et al., 2021; Wang; et al., 2015). In Arabidopsis, OST1 has been found to regulate ABA-induced stomatal closure through phosphorylating both the anion and cation channels of SLAC1 and POTASSIUM CHANNEL 1. Another mechanism that plants can positively regulate stomatal closure under ABA influence is by getting OST1 to form a complex with Brassinosteroid-Insensitive 1 (BRI1)-Associated Receptor Kinase 1 (BAK1) (Deng; et al., 2022; Shang; et al., 2016). However, these two research groups reported opposite effects of BAK1 on OST1’s activity. More investigation is required to validate whether BAK1-mediated phosphorylation of OST1 contributes positive or negative effects on OST1 activity. In addition to phosphorylation, ABA-induced production of nitric oxide (NO) in guard cells was reported to negatively regulate OST1 activity through S-nitrosoglutathione (GSNO) reductase (GSNOR) mediated S-nitrosylation; indicating that multiple modifications of OST1 may be required for a precise regulation of its activity in response to stress (Fig. 1).

Under drought conditions, plants can minimize transpiration water loss by synthesizing ABA. ABA can then act directly on guard cells to trigger potassium and anions efflux from guard cells as well as removing organic osmolytes to close stomatal aperture (Gong; et al., 2020a). ABA- and drought-induced stomatal closure are severely impacted in Arabidopsis *ost1* mutants (Mustilli; et al., 2002; Yoshida; Mogami; Yamaguchi-Shinozaki, 2014). *ost1* single mutants display no obvious developmental phenotypes other than impaired stomatal movement. However, *snrk2.2* *snrk2.3* *snrk2.6* triple mutant displays extreme insensitivity to ABA (Fujita; et al., 2009; Nakashima; et al., 2009). In Arabidopsis, SnRK2.2 and SnRK2.3 are mainly expressed in seeds and they possess similar functions in ABA inhibition of seed germination. The triple mutant also shows a significant reduction in its tolerance to drought that accompanies the suppression of ABA- and osmotic stress-induced genes with conserved ABA-responsive elements (ABREs) in their promoters (Fujita; Yoshida; Yamaguchi-Shinozaki, 2013; Zhang; et al., 2022). This evidence indicates that OST1 plays an important role in ABA-induced stomatal closure in response to drought.

## OST1 is a key mediator in cold stress

Asides from acting on guard cells, OST1 has been reported as a key protein kinase in plant response to cold stress (Ding; Yang, 2022). The negative regulator EGR2 (E GROW REGULATING 2) protein phosphatase inhibits OST1’s activation by binding to OST1 under normal conditions. Upon cold stress situation, the inhibition on OST1 by EGR2 is released and thus, OST1 becomes activated to positively regulate freezing tolerance in Arabidopsis (Ding; et al., 2019). A more recent study has revealed that PP2CG1 (PROTEIN PHOSPHATASE 2C G GROUP 1) phosphatase can interact with OST1 to inhibit its activity and negatively regulate the protein abundance of CBFs. Cold stress can inhibit OST1-PP2CG1 interaction and the cold-activated OST1 can phosphorylate PP2CG1 to further suppress its phosphatase activity, uncovering a phosphor/dephosphor-regulatory loop in plant cold stress response (Lv; et al., 2021). It is worth noting that the ABA or drought-induced activation of OST1 relies on ABA stimulation, ABA receptors and PP2C whereas the cold activation of OST1 is independent of ABA and ABA receptors (Chen; et al., 2020; Gong; et al., 2020b). OST1 acts upstream of the CBF (C-REPEAT BINDING FACTOR/DEHYDRATION-RESPONSIVE ELEMENT-BINDING PROTEIN 1) pathway and directly interacts with and phosphorylates the key TF ICE1 (Inducer of CBF Expression 1), which disrupts the interaction of ICE1 with the E3 ligase HOS1 and promotes its protein stability and transcriptional activity (Ding; et al., 2015).

Moreover, cold-activated OST1 can interact with or phosphorylate BTF3 (Basic Transcription Factor 3), BTF3L (BTF3-Like), and two U-Box type E3 ligases (PUB25 and PUB26). The BTF3s phosphorylation by OST1 can promote their interactions with CBF proteins and stabilize CBF proteins stability under cold conditions. While the phosphorylation of PUB25 and PUB26 by OST1 can target the negative regulator MYB15 upstream of CBFs for degradation (Ding; et al., 2018; Wang; et al., 2019). Therefore, these combined events help to increase the expression of CBF genes to help plants withstand cold stress. In addition to regulating CBF pathway, the rice OST1 homolog gene OsSAPK8 has been reported to phosphorylate and activate OsCNGC9 (CYCLIC NUCLEOTIDE-GATED Ca^2+^ channel) to induce calcium accumulation in the cytosol; further activating the expression of OsDREB1 expression to positively regulate chilling tolerance (Wang; et al., 2021). Interestingly, *At*OST1 can interact with and phosphorylate a Ca^2+^-permeable transporter *At*ANN1 (ANNEXIN1) to enhance its Ca^2+^ transport activity, thus revealing a cascade that links OST1-AtANN1 module to cold triggered Ca^2+^ signaling (Liu; et al., 2021).

## OST1 modulates ABA/drought mediated transcription in Arabidopsis and maize

Drought can trigger a complex range of responses in plants that can decrease their productivity and survival chances. These responses include stomatal closure, reduced turgor pressure, altered leaf gas composition, and decreased photosynthesis rates (Zhang; Zhao; Zhu, 2020). Recently, SnRK2.6 has been shown to cooperate with the Mediator subunit called CDK8 (cyclin-dependent kinase 8) in Arabidopsis to regulate stress response. It has been suggested that once drought and/or ABA signals are transmitted to SnRK2.6, this protein kinase can directly interact with RAP2.6 (RELATED TO AP2 6) to induce the transcription of several ABA-responsive genes containing GCC/DRE-motif such as RD29A and COR15A. The Mediator CDK8 physically interacts with RAP2.6 and facilitates the recruitment of RNA polymerase II to the promoters of RAP2.6 target genes and initiate downstream transcriptional changes (Chong; Guo; Zhu, 2020; Chong; Shi; Zhu, 2020; Zhu; et al., 2020). In maize, ZmOST1 is highly homologous to OST1. In response to ABA and water levels, ZmOST1 has been shown to enhance plant drought tolerance through regulating stomata closure to decrease water loss. Upon sensing stress signals, ZmOST1 mediates the efflux of anions from the guard cells to close off stomata (Wu; et al., 2019). Most recently, one study reported that SnRK2s including OST1 can phosphorylate RAF36 and RAF22 to promote their degradation; therefore alleviating their inhibitory roles in ABA signaling. Similarly, another study also indicated that exogenous ABA can activate OST1 to phosphorylate RAF22 and inhibits its activity, thereby preventing RAF22 to enhance the phosphatase activity of ABI1 (ABA Insensitive 1)(Kamiyama; et al., 2021; Sun; et al., 2022). These studies suggested that OST1 may form a dynamic network with RAF22/ARF36 and ABI1 to optimize plant growth and responses to ABA and drought.

## OST1 promotes plant shoot: root ratio during drought stress

Under a drought condition, plants can activate the essential SnRK2 members in the ABA signaling pathway including OST1 to manage their root growth and stress response (Chen; et al., 2022). It has been shown that ABA signaling can play an important role in reshaping the root architecture of plants during drought. Evidently, plants can choose and block a type of root and shoot development for drought adaptation. One suggested drought-survival approach is that plants restrain their lateral root and shoot growth, and augment their primary root growth so they can better acquire water from deep soil during drought (Gong; Yang, 2022). Another drought survival strategy that plants can undertake is to generate more lateral roots to obtain more water and nutrients. Root development during drought requires sucrose to be transported from shoots to roots and the transporters responsible for sucrose transport are SWEET (Sugars Will Eventually Be Exported Transporter) 11 and 12. ABA-responsive SnRK2 protein kinases play an essential role in root development during drought because they can phosphorylate Ser237 and Ser248 of SWEET11 and 12 to regulate root growth. SnRK2.2, 2.3 and 2.6 are first activated by ABA under drought stress, then they phosphorylate SWEET11 and 12 and promote homo- and hetero-oligomerization of SWEET11 and 12, thus forming a channel for sucrose diffusion from parenchymal cells to companion cells. As a result, sucrose is transported to roots, thus promoting root growth under drought stress. Additionally, phloem loading from leaf cells can be controlled by SWEET11 and 12 sucrose exporters. SWEET11 and 12 can mediate sucrose allocation and root growth associated with osmotic stress. Therefore, OST1 plays an important role in mediating sucrose transport from shoots to roots during drought. This finding reinforces the importance of OST1 as it involves with the activation of the ABA signaling pathway to help plants modulate their growth and development under stress.

## OST1 balances drought response and flowering in tomato

Tomato *SlOST1* has been revealed to help tomato adapt to drought and balance flowering (Chong; et al., 2022). It has been suggested that the loss of function of *SlOST1* results in drought hypersensitivity and late flowering under both normal and drought stress conditions. SlOST1 can interact with and phosphorylates the NAC-type TF SlVOZ1 (VASCULAR PLANT ONE-ZINC FINGER 1). The binding of SlOST1 with SlVOZ1 leads to increased protein stability and nuclear accumulation of SlVOZ1. Nucleus-localized SlVOZ1 can then directly bind to the *SFT* (*SINGLE FLOWER TRUSS*) promoter to stimulate tomato flowering in response to drought stress. ABA is also shown to enhance the phosphorylation, protein stability and nuclear translocation of SlVOZ1; thereby indicating the essential role of ABA in regulating SlOST1-mediated flowering under drought. Flowering in tomato is a crucial developmental stage that can be susceptible to environmental stress such as drought. SlOST1 can not only positively regulate drought tolerance but also flowering time under drought conditions. The SlOST1-SlVOZ1 module is hence suggested to play a critical role in balancing drought stress responses and flowering transition in tomato.

## Concluding remarks and future perspectives

Plants are constantly exposed to various environmental stimuli that can threaten their growth and productivity. Plants can adapt to their fluctuating environment via the accumulation of the phytohormone ABA and activate the ABA signaling pathway. Evidently, ABA plays a significant role in important biological processes such as growth, development and responses to various environmental stresses across different plant species.

SnRK2.6 or OST1 is an active Ser/Thr subclass III SnRK2 kinase that contains several autophosphorylation sites. During the absence or presence of very low ABA levels, PP2C can interact with OST1 and dephosphorylate OST1. When ABA is present, ABA receptors and ABA-bound PYR1/PYL/RCARs can recruit ABI1 from OST1. Activated OST1 can phosphorylate various TFs such as bZIP TFs and ABFs/ABRES, to induce ABA signaling. OST1 can also bind with other protein(s) to form a module and/or a dynamic network that regulates downstream gene expression in the nucleus which further influence stomatal closure, abiotic/biotic responses, seed germination, seedling growth, flowering, and root growth in plants. Therefore, OST1 not only serves as a core component in ABA signaling but controls plant growth and development as well.

Based on the current evidence we have about OST1, we can see that OST1 possesses various functional roles in plants as it can regulate stress response, stomata apertures, ROS production as well as plant growth and development. In fact, OST1 is a SnRK2 protein kinase that has roles beyond stress regulation. OST1 may have functional roles in other plant developmental stages such as fruit ripening that involves stress response activation (Jia; et al., 2022). Perhaps, we may want to explore OST1’s role in regulating fruit ripening and drought response for future research. Fruit ripening involves the accumulation of pigment, sugar, acid, aroma compounds, and many other substances. Fruit quality is affected by ethylene and osmotic stress. Additionally, abiotic stress factors such as drought and salinity can dramatically reduce the quality and yield of apple fruit. However, moderate drought can enhance apple fruit quality. It is possible that OST1 can also modulate fruit ripening process through phosphorylating downstream targets under drought conditions in an ABA-dependent manner. OST1 could be a target gene for agricultural improvement and we can get one step closer to developing an ideal plant species that withstands harsh environment by studying more on how OST1 can simultaneously mediate stress signaling pathways and important biological processes.

## Data Availability

Not applicable.

## Abbreviations

OST1: Open Stomata 1
SnRK2: Sucrose non-fermenting 1 (SNF1)-Related protein Kinase 2
ABA: Abscisic acid
PYR/PYL/RCAR: Pyrabactin Resistant/Pyrabactin Resistant-Like/Regulatory Component of ABA Responses
TOR: Target of Rapamycin
RAFs: Raf (Rapidly Accelerated Fibrosarcoma)-like kinases
GHR1: GUARD CELL HYDROGEN PEROXIDE-RESITANT 1
SLAC1: SLOW ANION CHANNEL-ASSOCIATED 1
KAT1: K^+^ Inward Rectifying Channel
PTMs: Post-Translational Modifications
ABREs: ABA-Responsive Elements
EGR2: Clade-E Growth-Regulating 2
CBF: C-REPEAT BINDING FACTOR/DEHYDRATION-RESPONSIVE ELEMENT-BINDING PROTEIN 1
ICE1: Inducer of CBF Expression 1
PP2CG1: PROTEIN PHOSPHATASE 2C G GROUP 1
BTF3: Basic Transcription Factor 3
CNGC9: CYCLIC NUCLEOTIDE-GATED Ca^2+^ channel
CDK8: Cyclin-Dependent Kinase 8
RAP2.6: RELATED TO AP2 6
ABI1: ABA Insensitive 1
SWEET: Sugars Will Eventually Be Exported Transporter
VOZ1: VASCULAR PLANT ONE-ZINC FINGER 1
SFT: SINGLE FLOWER TRUSS
